# Transradial Subclavian Artery Stenting With Proximal Vertebral Artery Protection Using a Sheathless Balloon Guiding Catheter: A Case Report

**DOI:** 10.7759/cureus.101315

**Published:** 2026-01-11

**Authors:** Shin Nemoto, Yuya Ogura, Ririko Takeda, Makoto Nakane

**Affiliations:** 1 Department of Neurosurgery, Teikyo University Hospital - Mizonokuchi, Kawasaki, JPN

**Keywords:** balloon guiding catheter, cerebral embolic protection, endovascular stenting, sheathless technique, subclavian artery stenosis, transradial approach, vertebral artery protection

## Abstract

Subclavian artery (SCA) stenting is an established treatment for symptomatic stenosis; however, the optimal strategy for embolic protection of the vertebral artery (VA) remains controversial, particularly in cases with preserved antegrade VA flow. We report a case of symptomatic left SCA stenosis treated using proximal VA protection with a sheathless balloon-guiding catheter (BGC) via a transradial approach. A 77-year-old man with a history of cerebral infarction and coronary artery disease presented with dizziness. Preoperative imaging demonstrated severe stenosis of the left SCA adjacent to the VA origin with markedly reduced but preserved antegrade VA flow, indicating a potential risk of vertebrobasilar embolization during intervention. Endovascular stenting was performed through left transradial access. To achieve proximal embolic protection while minimizing access site trauma, an 8 Fr GBC was introduced using a sheathless technique and inflated at the VA origin to achieve temporary flow arrest during lesion crossing and stent deployment. After aspiration, normal antegrade flow in the VA was restored. The procedure was completed without neurological complications, and postoperative imaging confirmed improved VA flow. Proximal VA protection using a sheathless BGC via a transradial approach represents a feasible and safe strategy for SCA stenting in selected patients with preserved antegrade VA flow, particularly when distal protection devices are technically challenging.

## Introduction

Subclavian artery (SCA) stenosis represents an important cause of upper extremity ischemia and vertebrobasilar insufficiency. Endovascular stenting has been widely adopted as the preferred revascularization strategy owing to its high technical success rate and lower procedural morbidity compared with surgical reconstruction [1]. Nevertheless, cerebral embolic complications, particularly those involving the vertebral artery (VA), remain a critical concern during endovascular intervention.

The role of routine VA protection during SCA stenting remains a subject of debate. In patients with complete subclavian steal syndrome, retrograde VA flow may theoretically mitigate cerebral embolic risk by directing embolic material toward the upper extremity [2]. Conversely, in cases with preserved antegrade VA flow, embolic debris generated during lesion crossing, balloon angioplasty, or stent deployment may directly enter the posterior circulation, thereby increasing the risk of vertebrobasilar ischemic events.

Several embolic protection strategies have been described, including unprotected stenting, distal filter-based protection, and proximal balloon occlusion [3-5]. Distal protection devices require lesion traversal prior to deployment, a step that may itself increase embolic risk and can be technically challenging in the presence of vessel tortuosity or unfavorable anatomy. Proximal balloon occlusion offers the theoretical advantage of establishing cerebral protection before lesion crossing [6]. However, reports describing this strategy using a transradial sheathless approach with a balloon-guiding catheter (BGC) remain scarce.

The present report describes the successful treatment of symptomatic left SCA stenosis with preserved antegrade VA flow using proximal VA protection with a sheathless BGC via a transradial approach, highlighting the hemodynamic rationale and technical considerations underlying this strategy.

## Case presentation

**Figure 1:**
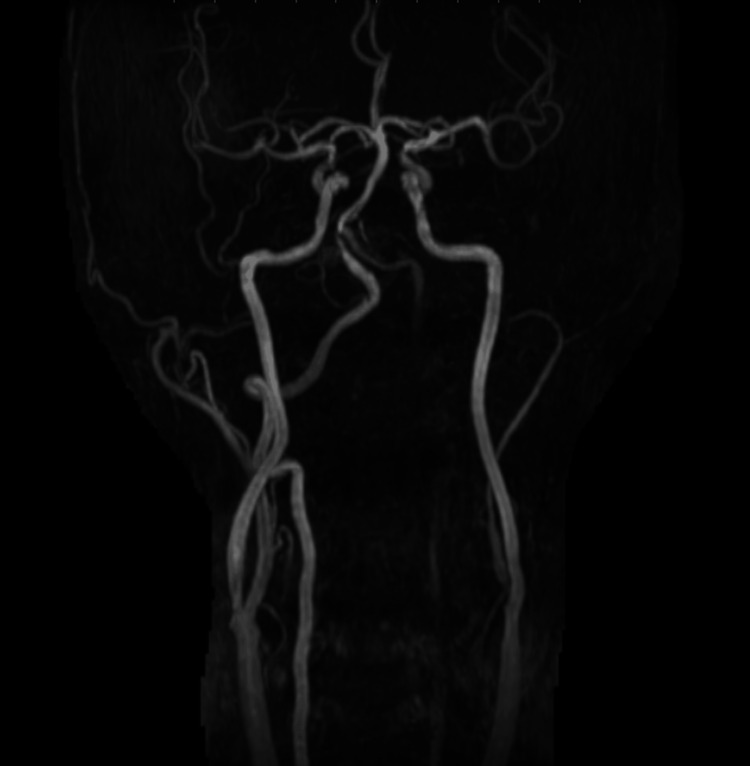
Preoperative magnetic resonance angiography (MRA) of the cervical vessels demonstrating reduced signal intensity in the left vertebral artery, suggestive of decreased antegrade flow

A 77-year-old man with a history of cerebral infarction and coronary artery disease presented with dizziness. Preoperative magnetic resonance angiography (MRA) demonstrated reduced signal intensity in the left VA, suggestive of decreased antegrade flow (Figure 1).

**Figure 2:**
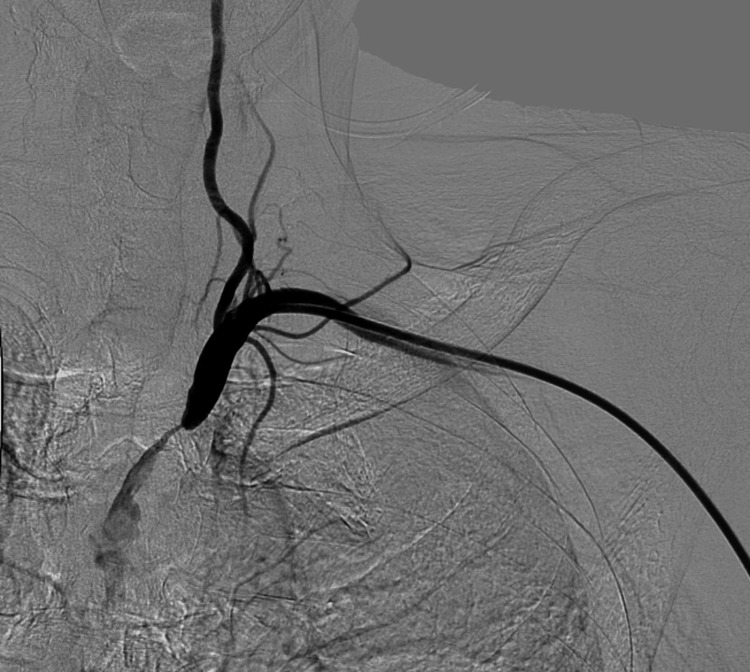
Preprocedural digital subtraction angiography demonstrating severe eccentric stenosis of the left subclavian artery with residual antegrade flow into the vertebral artery

Digital subtraction angiography (DSA) via a left transradial approach confirmed severe eccentric stenosis of the SCA. Although flow was sluggish, residual antegrade flow into the VA was clearly identified, indicating a high risk of vertebrobasilar embolization during intervention (Figure 2).

**Figure 3:**
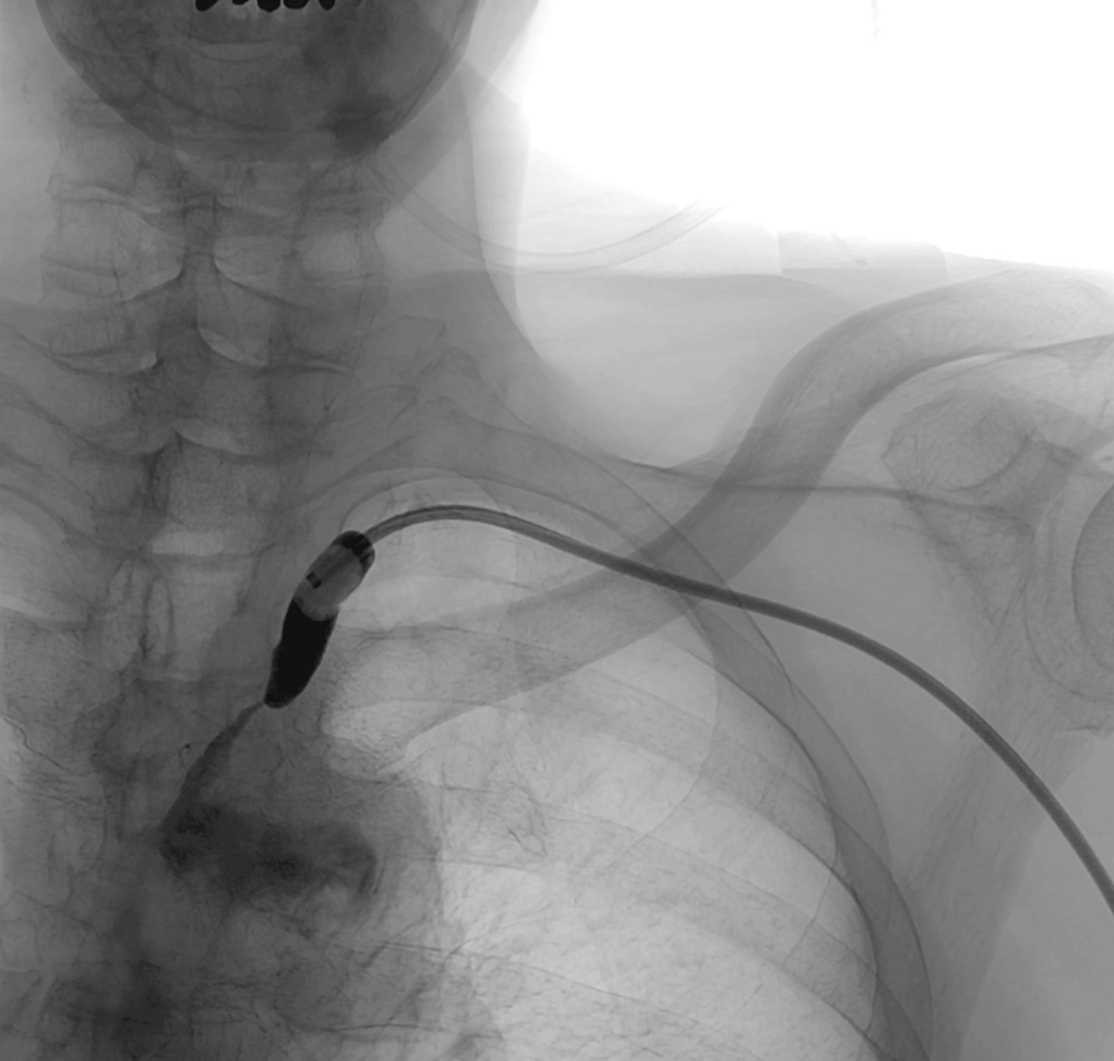
Intraprocedural angiography demonstrating complete flow arrest in the vertebral artery after inflation of the balloon guiding catheter at its origin

To achieve proximal embolic protection while minimizing access site trauma, an 8 Fr BGC (Optimo Flex; Tokai Medical Products, Aichi, Japan) was introduced into the VA origin using a sheathless technique. Upon inflating the BGC balloon, complete flow arrest in the VA was confirmed (Figure 3).

**Figure 4:**
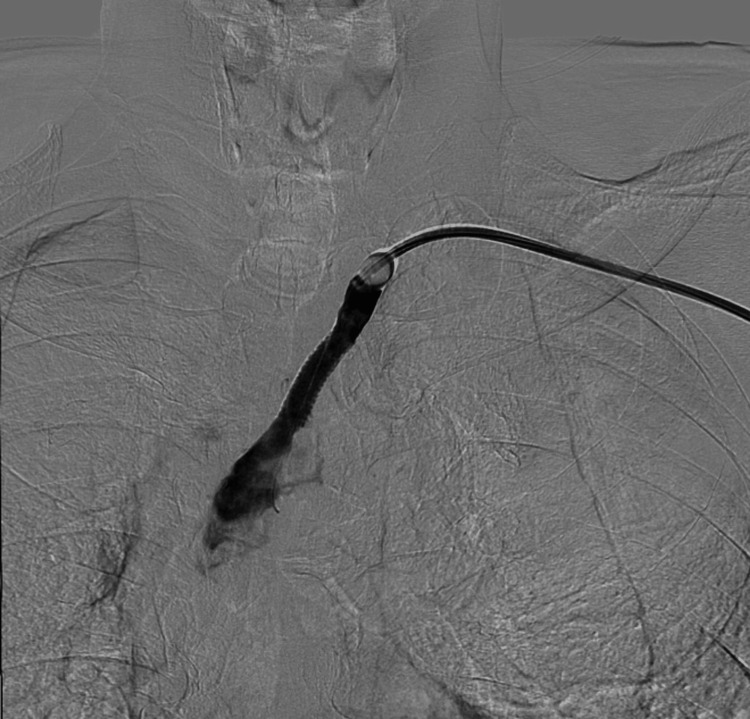
Post-stenting angiography under balloon-guiding catheter protection

Under this protection, the lesion was crossed, and a stent was successfully deployed. Angiography performed immediately after stent deployment, while the BGC remained inflated, confirmed that flow arrest in the VA was still maintained, preventing any debris from migrating distally (Figure 4).

**Figure 5:**
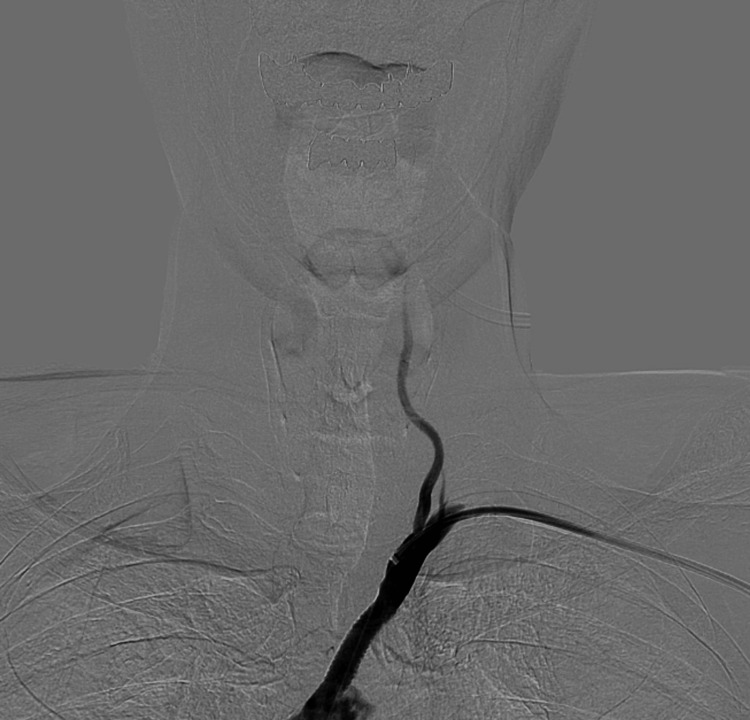
Final angiography showing restoration of the subclavian artery lumen and antegrade vertebral artery flow

Following thorough aspiration of potential debris, the BGC balloon was deflated. Final angiography showed successful restoration of the SCA lumen and brisk physiological flow into the VA (Figure 5).

**Figure 6:**
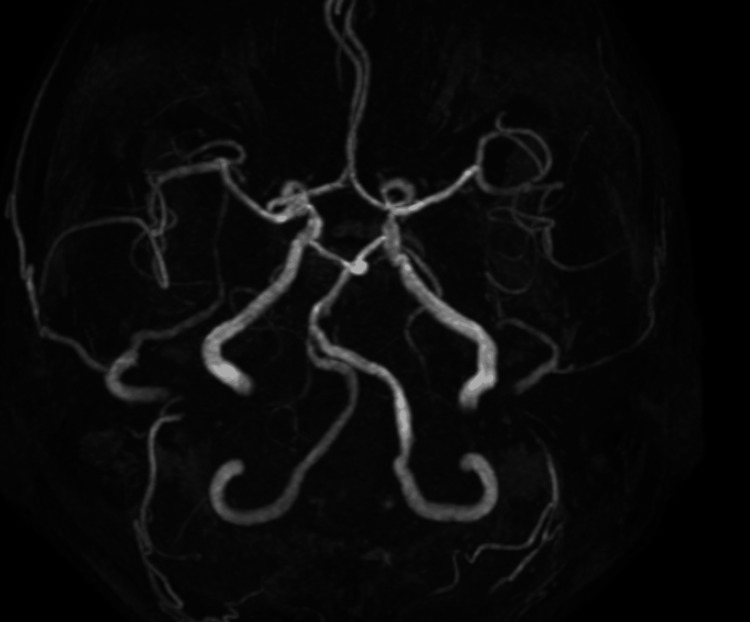
Postoperative magnetic resonance angiography demonstrating improved flow in the left vertebral artery

The procedure was completed without neurological complications. Postoperative MRA demonstrated marked improvement in the signal intensity of the left VA, consistent with successful revascularization (Figure 6).

## Discussion

This case underscores two key aspects in the management of high-risk SCA stenosis: the importance of hemodynamic assessment in determining the need for VA protection and the technical advantages of a transradial sheathless approach employing a BGC.

Embolic protection strategies during SCA stenting should be individualized based on VA flow dynamics. In the setting of complete subclavian steal syndrome, retrograde VA flow may reduce the likelihood of cerebral embolization [2]. In contrast, when antegrade VA flow is preserved, as in the present case, embolic material generated during endovascular manipulation may directly access the posterior circulation, thereby conferring an increased risk of vertebrobasilar stroke. Proximal BGC protection offers distinct advantages. Flow arrest is established before lesion crossing, unlike distal filter devices that require traversing the stenosis before protection is achieved.

Recently, Yamazaki et al. reported an advanced "balloon switching technique" for SCA stenting using an 8 Fr sheathless BGC via transradial access [7]. Their method involves alternating balloon inflation between the stent delivery system and the BGC to induce an intentional subclavian steal phenomenon, which effectively flushes debris away from the VA. While our procedure did not utilize this specific switching maneuver, our successful outcome reinforces the fundamental safety and utility of proximal BGC protection in SCA stenting.

Access strategy represents another critical determinant of procedural safety. In addition, a peel-away sheath technique has been reported as a safe and reproducible method for sheathless transradial delivery of an 8 Fr BGC using commonly available devices, further expanding the feasibility of transradial access in neuroendovascular practice [8]. Large lumen-guiding systems facilitate stable device delivery and effective aspiration but conventionally require femoral or brachial access, which is associated with higher rates of access site complications. The use of a sheathless technique reduces the effective outer diameter of the access system, thereby permitting introduction of an 8 Fr BGC via the radial artery. This approach integrates robust embolic protection with the established safety profile of transradial access and aligns with the principles of slender endovascular intervention [9].

Several limitations warrant consideration. This report describes a single clinical experience, and the generalizability of the technique cannot be assumed. Temporary VA occlusion may not be tolerated in patients with insufficient collateral circulation or contralateral VA hypoplasia, underscoring the importance of careful preprocedural assessment of posterior circulation hemodynamics. Furthermore, successful implementation of this strategy requires familiarity with sheathless systems and BGC techniques, which may limit widespread adoption.

## Conclusions

Proximal VA protection using a BGC may represent a safe and effective strategy for subclavian artery stenting in carefully selected patients with preserved antegrade VA flow. When combined with a transradial sheathless approach, this technique may facilitate minimally invasive intervention while maintaining a high level of embolic safety and may serve as a potential alternative in cases where distal protection is technically challenging.
